# Dangerous parking deck

**DOI:** 10.1136/tsaco-2019-000327

**Published:** 2019-05-28

**Authors:** David V Feliciano

**Affiliations:** Department of Surgery, University of Maryland School of Medicine, Baltimore, Maryland, USA

**Keywords:** lower extremity trauma, arterial repair, tibia fracture, shunt

## History

A 53-year-old woman standing next to a car in a parking deck was struck by another car when the driver lost control. The patient’s right knee and leg were crushed between the bumpers of both cars. The patient was ‘fortunate’ in that a Level I trauma center was only six blocks away.

## Examination

On arrival in the trauma room, the patient was awake and alert with a heart rate of 120 beats per minute and a systolic blood pressure of 90 mm Hg. The right lower extremity was mangled with a dislocation of the knee, large wound in soft tissue with oozing behind the knee, and disrupted muscles in the exposed posterior compartments. No arterial pulses were present in the right foot, but there was sensation and some weak dorsiflexion and plantar flexion in the ankle joint.

## Question

The most appropriate first step in the management of this patient in addition to resuscitation is:

- CT arteriography.
- Administer unfractionated heparin.
- Obtain consent for amputation.
- Move patient to operating room.

## Management

The patient was moved to the operating room to control bleeding from disrupted soft tissue, assess the magnitude of injuries to the right knee and leg and to obtain X-rays of the same. After preparation of the skin from the umbilicus to the toenails bilaterally, the right foot was placed in a plastic bag to allow for later observation of skin color changes and palpation of pedal pulses. The remainder of both lower extremities was draped in the usual fashion.

As there was no major arterial hemorrhage from disrupted soft tissues, X-rays of the right knee and leg were performed. A Grade IIIC open dislocation of the right knee joint was confirmed, and there was a Grade II open transverse fracture of the right tibia, as well. A distal medial right popliteal incision was made beyond the aforementioned soft tissue defect to allow for better exposure of the neurovascular bundle. The popliteal artery and vein and the anterior tibial artery and vein were all avulsed with the ends thrombosed, whereas the tibial (medial popliteal) nerve was intact. The common peroneal (lateral popliteal) nerve was not readily visualized, but the soft tissue in the area of the nerve was intact.

## Question

The most appropriate management of this mangled right lower extremity would be:

- Above knee amputation.
- Insert intraluminal vascular shunts.
- Insert saphenous vein grafts.
- Fixator to knee dislocation

## Management

The patient was now hemodynamically stable, had some neurological function in the right ankle and foot, had fixable orthopedic injuries and cold ischemia in the right leg and foot. A decision was made to attempt salvage of the mangled right lower extremity.

The orthopedic surgery service first reduced the dislocation of the right knee joint. The trauma surgery service then performed dissection in the distal right popliteal area through the medial exposure. The proximal and distal ends of the avulsed and thrombosed popliteal artery were controlled, debrided and flushed. A #4 Fogarty balloon catheter was passed proximally and distally into the popliteal artery with no return of clot. ‘Regional’ heparinization was administered with the injection of 20 mL of a solution of 50 units of unfractionated heparin/mL normal saline into each end of the artery and each end of the vein. #12 Fr intraluminal temporary vascular shunts were then inserted into the ends of the popliteal artery and vein and fixated in place with 2–0 silk ties. Doppler-audible right pedal pulses were confirmed. The avulsed and thrombosed ends of the anterior tibial artery and vein were ligated.

As the patient remained hemodynamically stable with a body temperature >35°C, the orthopedic surgery service scrubbed back in. An intramedullary rod was inserted to stabilize the fracture in the right mid-tibia, whereas an external fixator was placed to stabilize the previously dislocated right knee.

The trauma surgery service then scrubbed back in and excised a long segment of the greater saphenous vein from the anteromedial left thigh. A reversed saphenous vein interposition graft was inserted into the defect in the distal right popliteal artery using 6–0 polypropylene sutures on both end-to-end anastomoses. Right pedal pulses were immediately palpable through the plastic bag over the right foot after the vascular clamps were removed. A non-reversed saphenous vein interposition graft was then inserted into the defect in the proximal popliteal vein in the same fashion. Fasciotomies of the anterior and peroneal muscle compartments of the right leg were performed through a 30 cm long skin incision two fingerbreadths superior to the right fibula laterally. Finally, contused and devascularized muscle in the right posterior calf muscle compartments was debrided back to bleeding tissue. Dry mesh gauze was then packed into the open wound in the right posterior popliteal area. Operative time for this first salvage procedure was 9 hours, and 12 units of packed red blood cells and two units of fresh frozen plasma were administered.

The patient underwent reoperations on postoperative days 1, 6, 7, 12, 13, 15 and 27. The first five reoperations were for pack changes in the open wound (#4) or for bleeding from granulation tissue (#1). Split-thickness skin grafts were applied to the right posterior popliteal open wound and to the right lateral leg fasciotomy site on postinjury day #27. The skin grafts had 100% healing, and the patient was discharged home on postinjury day #38.

**Figure 1:**
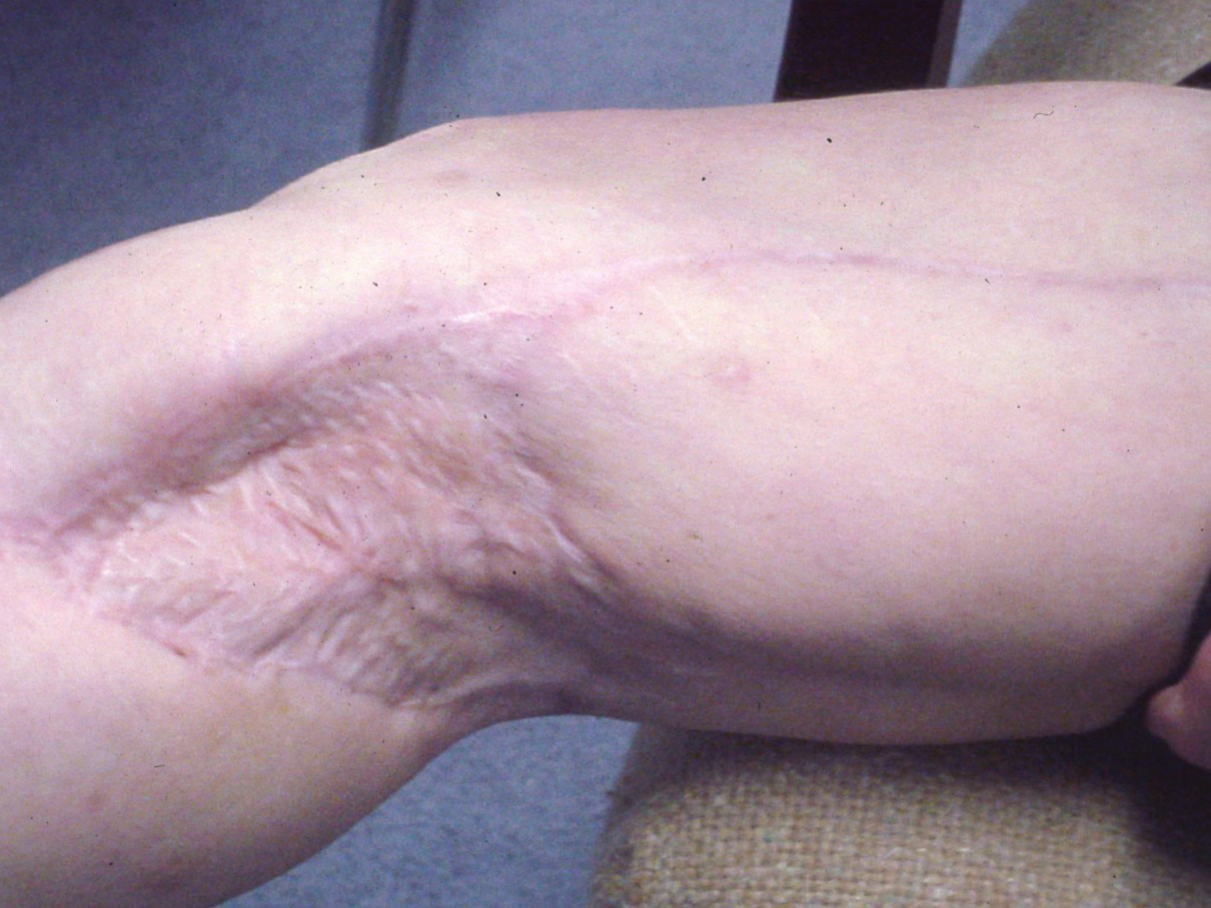
Healed split-thickness skin graft in right posterior popliteal area at 8 months and 3 weeks after injury.

**Figure 2:**
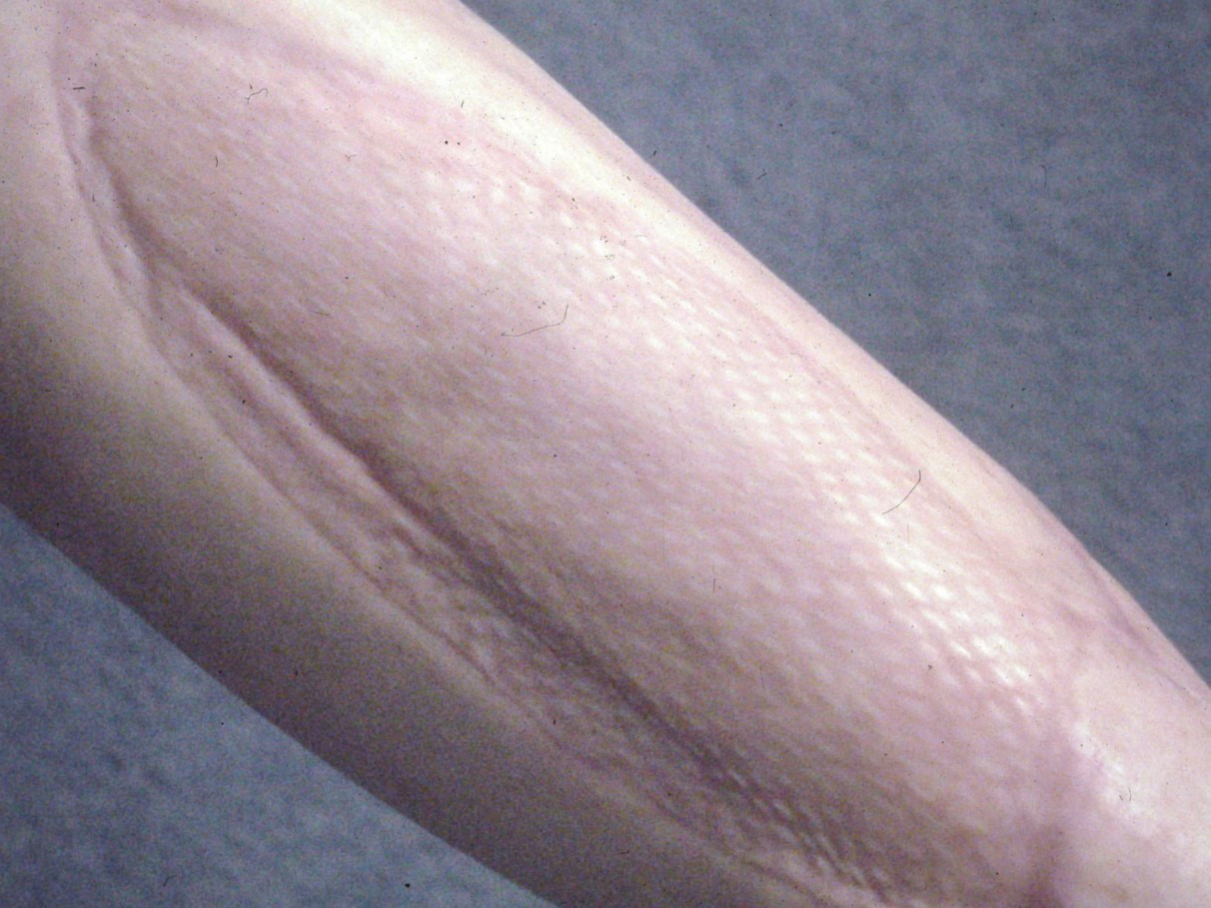
Healed split-thickness skin graft on right lateral leg fasciotomy site at 8 months and 3 weeks after injury.

After removal of the fixator and extensive physical therapy, the patient was walking with a cane when seen in the clinic 8 months and 3 weeks after injury (figures 1 and 2).

**Figure 3:**
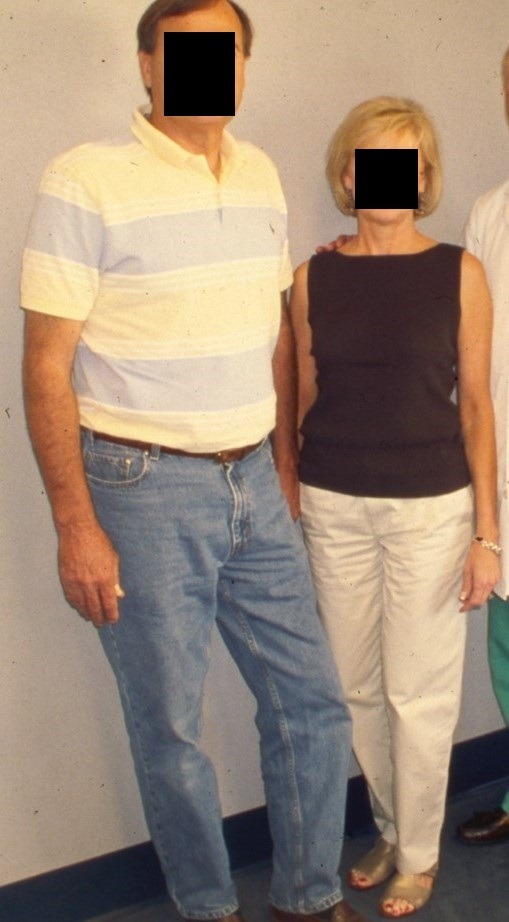
Patient with intact right lower extremity 46 months after injury.

At 46 months after injury, the patient continued to ambulate well and was encouraged to cease use of the cane (figure 3). Her major complaint was ‘aching’ in the formerly mangled right knee and leg when the weather changed.

## Discussion

Mangled extremities result from a high energy transfer or crush resulting in some combination of injuries to artery, bone, soft tissue, nerve, and/or tendon (adapted from Feliciano and Rasmussen^1^).

All are Gustilo IIIC fractures or beyond, and 75% result from motorcycle or motor vehicle crashes, falls or motor vehicle-pedestrian crashes.^2 3^ For motor vehicle and auto-pedestrian crashes, it is helpful to remember that the kinetic energy transfer that occurs in a collision with an automobile bumper at 20 mph is 100 000 ft-lbs and this is 50 times greater than that from a high velocity rifle wound.^4^

The problems associated with managing patients with mangled extremities are either acute or, if salvage is completed, chronic (table 1).

**Table 1:** Acute and chronic problems associated with a mangled extremity

Tissue	Acute	Chronic
Artery	Ischemia; loss of collaterals	Graft occlusion
Bone	Bone loss	Infection; instability
Nerve	Loss of function	Need nerve grafts
Soft tissue	Open wound	Need flap coverage
Tendon	Loss of movement	Need tendon transfer

The most important decision when managing a patient with a mangled extremity is whether an immediate amputation or initiation of a series of salvage operations should be performed. One way to approach this decision is to attempt to answer the first three of the following four questions in sequence:

1. If the patient’s life is in danger from blood loss or associated injuries, should the mangled extremity be amputated immediately?
2. If the patient is hemodynamically stable, should salvage of the mangled limb be attempted (ie, is there intact neurological function in the foot or hand, is there a reconstruction option for the open fracture, and is there an option for coverage of all open wounds?)?
3. If salvage is to be attempted, what is the sequence of repairs?
4. If salvage fails (sepsis, chronic infection, drainage, pain, disability), when should amputation of the repaired extremity be performed?

There is reasonable consensus that if either of the following factors is present in an injured lower extremity, immediate amputation rather than initiation of salvage operations is recommended:

1. Loss of arterial inflow for >6 hours, particularly in the presence of a crush or avulsion injury that disrupts collateral vessels.^5 6^
2. Disruption of the posterior tibial nerve behind the knee.

Relative indications for immediate amputation with Grade IIIC tibial fractures have been described, as well, especially if two of the following are present:

1. Serious associated polytrauma.
2. Severe ipsilateral foot trauma.
3. Anticipated protracted course to obtain soft tissue coverage.
4. Need for extensive tibial reconstruction.^5 7 8^

The patient with a mangled extremity should have resuscitation and control of bleeding in the emergency room (pressure dressing or tourniquet) and any critical non-extremity imaging to rule out or confirm associated injuries. In the operating room, the sequence of evaluation and management is as follows:

1. Continue resuscitation if patient hypotensive and obtain baseline thromboelastography.
2. Assess sensation and motor function in the foot or hand of the mangled extremity.
3. X-ray the mangled extremity.
4. Assess arterial inflow by physical examination, Doppler device or percutaneous surgeon-performed arteriogram.
5. If no arterial inflow and salvage possible, insert temporary intraluminal arterial (and venous, if needed) shunt.
6. Classify bony/soft tissue and other injuries in the mangled extremity with attending surgeons, fellows, or senior residents on orthopedic and plastic surgery services.
7. Visualize major nerves through open wound.
8. If needed to help make decision on attempted salvage versus immediate amputation, calculate magnitude of injury using a scoring system.
9. Trauma vascular, orthopedic and plastic surgery attending surgeons, fellows or senior residents make a decision on salvage versus amputation.
10. If immediate amputation is indicated, have a designated family member put on a scrub suit and come into the operating room for a visual explanation of the decision.^9^

After an initial operation for salvage, it is important to explain to the family that a total of four operations (at least) will be needed, that there has been a historic 30% to 50% incidence of late amputations after attempted salvage and that chronic disability is likely to be present even if salvage is successful.^10^

A comprehensive analysis of the results of attempted salvage versus immediate amputation is beyond this case discussion, but there are helpful data available.

The Lower Extremity Assessment Project has documented that 1-year follow-up is adequate to assess results and that functional outcomes are equivalent at 2 years and 7 years for salvage and amputation.^11 12^ In contrast, the Military Extremity Trauma Amputation/Limb Salvage study documented that military personnel who underwent amputation had better functional outcomes.^13^
